# Cardiopulmonary Exercise Testing in Elite Athletes: Rethinking Sports Classification

**DOI:** 10.3390/jcm14134655

**Published:** 2025-07-01

**Authors:** Maria Rosaria Squeo, Armando Ferrera, Sara Monosilio, Alessandro Spinelli, Viviana Maestrini, Federica Mango, Andrea Serdoz, Domenico Zampaglione, Roberto Fiore, Antonio Pelliccia, Giuseppe Di Gioia

**Affiliations:** 1 Institute of Sports Medicine and Science, National Italian Olympic Committee, Largo Piero Gabrielli, 1, 00197 Rome, Italy; armando.ferrera95@gmail.com (A.F.); sara.monosilio@uniroma1.it (S.M.); alessandro.spinelli1@gmail.com (A.S.); viviana.maestrini@uniroma1.it (V.M.); federicamango.md@gmail.com (F.M.); andreaserdoz@gmail.com (A.S.); domzamp1@gmail.com (D.Z.); roberto.fiore@mail.com (R.F.); antonio.pelliccia@coni.it (A.P.); dottgiuseppedigioia@gmail.com (G.D.G.); 2 Department of Clinical, Internal, Anesthesiologic and Cardiovascular Sciences, Sapienza University of Rome, Piazzale Aldo Moro, 5, 00185 Rome, Italy; 3 Department of Movement, Human and Health Sciences, University of Rome “Foro Italico”, Piazza Lauro De Bosis, 15, 00135 Rome, Italy

**Keywords:** sports cardiology, cardiopulmonary exercise test, sports medicine, elite athletes

## Abstract

**Background**: ESC sports classification in 2020, based on cardiac morphological adaptations, may not fully reflect also the variations in functional parameters of athletes. This study aims to characterize CPET-derived physiological parameters in elite athletes according to the ESC classification and evaluate whether this morphological classification also corresponds to a functional categorization. **Methods**: Elite athletes underwent pre-participation screening before the 2023 European Games and 2024 Olympic Games. Athletes were classified into four categories (skill, power, mixed and endurance). CPET was performed on a cycle ergometer using a ramp protocol, with measurements of VO_2_ max, heart rate, power output and ventilatory efficiency. **Results**: We enrolled 1033 athletes (46.8% females; mean 25.6 ± 5.2 years old) engaged in skill (14.1%), power (33.2%), mixed (33.3%) and endurance (19.4%) disciplines. O_2_ pulse showed an incremental significant increase (*p* < 0.0001) among sport categories (skill 14.9 ± 3.8 mL/beat; power 17.5 ± 4.6 mL/beat, mixed 19 ± 4.3 mL/beat and endurance 22.7 ± 5.8 mL/beat). The lowest ${\dot{V}O}_{2}$max was observed in skill disciplines (36.3 ± 7.9 mL/min/kg) whilst endurance ones showed the highest values (52.4 ± 9.7 mL/min/kg) (*p* < 0.0001). ${\dot{V}O}_{2}$max was higher in power compared to mixed (42 ± 7.7 mL/min/kg vs. 40.5 ± 5.8 mL/min/kg, *p* = 0.005) disciplines with an overlapping amount between some mixed and power disciplines. No differences were found for VE max (*p* = 0.075). **Conclusions**: Our study provided values of CPET parameters in elite athletes. Significant differences in CPET parameters were observed among different sports disciplines, with endurance athletes showing the highest absolute and relative values in all parameters. An overlap amount was noted between mixed and power categories, especially for relative maximal oxygen consumption.

## 1. Introduction

Over time, sports classification has been based on physiologic characteristics of the exercise (i.e., dynamic, static) [1,2] until 2020, when the European Society of Cardiology (ESC) guidelines on sports cardiology and exercise introduced a new sport classification, closer to cardiologists’ understanding, based on the presence and type of cardiac remodeling, comprising four major classes of sports disciplines (i.e., skill, power, mixed and endurance) [3]. However, this classification does not account for the actual functional parameters of athletes, potentially limiting its applicability in sports medicine.

Cardiopulmonary exercise testing (CPET) is a comprehensive tool to assess the integrated function of the cardiovascular, pulmonary, vascular and musculoskeletal systems during exercise. Its application extends from performance optimization in athletes to clinical evaluation of those with known or suspected cardiopulmonary conditions. While CPET is widely used in the general population, its application in elite athletes still remains less explored, particularly concerning different sports categories. Indeed, elite athletes exhibit distinct physiological adaptations depending on their specific sport, necessitating tailored assessment criteria [4,5].

This study aims to characterize key CPET-derived physiological parameters in elite athletes divided into different sports disciplines according to the current ESC classification and to assess whether the morphological classification proposed by the ESC also reflects a functional categorization which incorporates both morphological and functional aspects.

## 2. Materials and Methods

The Institute of Sport Medicine and Science in Rome, affiliated with the Italian National Olympic Committee, is tasked with conducting medical evaluations of athletes selected for relevant and international competitions such as the Olympic, European and Mediterranean Games. The present study was approved by the Ethics Committee of Sapienza University of Rome and by the internal Review Board of the Institute of Sports Medicine and Science on 25 September 2024, with code 0851/2024. All athletes included in this study were fully informed of the types and nature of the evaluation and signed the consent form, pursuant to Italian Law. All clinical data assembled from the study population are maintained in an institutional database. These procedures comply with the World Medical Association’s Code of Ethics (Declaration of Helsinki).

For this study, we enrolled 1033 elite (Olympic) athletes who were evaluated for pre-participation screening before the Krakow 2023 European Games and Paris 2024 Olympic Games. The athletes underwent a thorough, multidisciplinary pre-participation screening, which included a complete physical exam, extensive blood tests, basal electrocardiography (ECG), transthoracic echocardiography (TTE) and a cardiopulmonary exercise test (CPET). All athletes enrolled in our study were elite athletes and, according to the current guidelines, trained for more than 10 hours per week [3].

Athletes participated in 42 different disciplines, divided into skill, power, mixed and endurance categories according to European classification and previous studies [3,6,7,8,9]. For sports disciplines not categorized within the ESC classification, we used the COCIS Italian Guidelines [10] to classify them, and in case of a different classification of the same discipline between European and Italian Guidelines, priority was given to the ESC classification. Athletes participated in the following sports disciplines: Archery, skeet shooting, target shooting, golf, park and street skateboarding, equitation, table tennis and sailing were classified as skill disciplines. Moreover, weightlifting, diving, synchronized swimming, taekwondo, athletics (<800 mt), boxing, artistic gymnastics, judo, Greek–Roman wrestling, climbing, Muai Thai, surfing, swimming (<400 mt), kickboxing, karate and BMX and mountain biking were grouped into power disciplines. Then, volleyball, beach volleyball, basketball, rugby, water polo, fencing, tennis, paddle tennis, beach soccer, sporting dancing, rhythmic gymnastics and badminton were included in mixed disciplines, and, finally, canoeing, rowing, marathon swimming, pentathlon, marathon running, cycling and triathlon were classified as endurance sports.

Anthropometric measurements were obtained, with body composition and percentage of body fat determined utilizing bioelectric impedance analysis (BIA101 Quantum, Akern, Pisa, Italy) employing a constant sinusoidal current at a frequency of 50 kHz and an intensity of 400 μA. Height and weight were recorded for each subject, and body mass index (BMI) was computed as weight (in kilograms) divided by height (in meters) squared. Body surface area (BSA) was calculated using the Mosteller formula [11].

A standard 12-lead ECG was conducted with the subject in a supine position, and interpretation was performed in accordance with international criteria for ECG interpretation in athletes [12]. Blood pressure was assessed via non-invasive brachial cuff measurement while in a supine position at rest, according to European Society of Cardiology guidelines [13], concurrently with the acquisition of apical views by TTE, prior to the execution of CPET [13].

### 2.1. Cardiopulmonary Exercise Test

We conducted CPET on a cycle ergometer (COSMED, Rome, Italy). The protocol included a one-minute rest, a two-minute warm-up without any load and subsequent increments of 15-20-25-30 Watts with a ramp protocol, depending on gender and sports discipline, until exhaustion [14]. Continuous ECG monitoring and recording (Quark T12x, COSMED) was obtained during the warm-up, exercise and subsequent recovery period, which lasted for five minutes. Additionally, we utilized a breath-by-breath metabolimeter (Quark CPET; COSMED) to measure oxygen consumption and carbon dioxide production throughout the entire cardiopulmonary assessment.

We recorded the following parameters at the peak:

1. ${\dot{V}O}_{2}$ in absolute (mL/min) and relative (mL/min/kg) values;
2. Power (expressed in Watts);
3. Heart rate (HR), bpm;
4. VCO_2_ (mL/min);
5. Respiratory quotient (RQ);
6. Oxygen Pulse (${\dot{V}O}_{2}$/HR).

When reaching both the lactate threshold and respiratory compensation threshold, measurements were conducted for the following:

7. Power (expressed in Watts);
8. ${\dot{V}O}_{2}$ (mL/min);
9. The slope of work efficiency (${\dot{V}O}_{2}$/watts).

Moreover, VE/CO_2_ was measured with the exclusion of the data beyond the ventilatory compensation point.

Lactate threshold was measured according to current guidelines. In particular, in our study, it is defined by the following events, all of which occur roughly simultaneously: the ${\dot{V}O}_{2}$ at which VE/${\dot{V}O}_{2}$ and PetO_2_ reach a minimum and thereafter begin to rise consistently, coinciding with an unchanged VE/VCO_2_ and PetCO_2_ [15].

### 2.2. Statistical Analysis

Categorical variables were expressed as absolute numbers and percentages (shown in parentheses), and group comparisons were conducted using either Fisher’s exact test or the Chi-square test, depending on suitability. The distribution of continuous variables was evaluated for normality; variables with normal distribution were presented as mean ± standard deviation (SD). For between-group comparisons of normally distributed continuous variables, independent-sample Student’s *t*-tests were applied. When assessing differences across multiple groups, the Dunn test with pairwise comparisons was employed. A *p*-value < 0.05 was considered indicative of statistical significance. All analyses were conducted using SPSS software, version 29 (SPSS Inc., Chicago, IL, USA).

## 3. Results

We enrolled 1033 elite athletes (Olympic and probable Olympic), 483 females (46.8%), with a mean age of 25.6 ± 5.2 years old, mostly Caucasians (47 Afro-Caribbean, 4.5%) with a mean BMI of 23.2 ± 2.9 kg/m^2^.

Overall, 5 athletes (0.5%) had hypertension under chronic pharmacological treatment, 2 athletes (0.2%) had insulin-dependent type I diabetes, 87 (8.4%) were active smokers, 25 (2.4%) had familiarity with sudden cardiac death (SCD) and 338 (32.7%) had a family history of cardiovascular diseases (CVDs). Athletes participated in a wide range of sports disciplines (42) divided into four categories according to the 2020 ESC classification: skill, 146 (14.1%); power, 343 (33.2%); mixed, 344 (33.3%; and endurance, 200 (19.4%).

In Table 1 are listed principal clinical and anthropometric differences among the main sport categories. Athletes practicing power disciplines were younger compared to the remaining categories (*p* < 0.0001); a higher prevalence of Afro-Caribbean athletes (7.9%, *p* = 0.0008) compared to skill disciplines (100% Caucasians athletes) and to mixed sports (3.5%, *p* = 0.013) was observed. A similar prevalence of female athletes was found between the groups (*p* = 0.218) but significant anthropometric differences were noted with mixed-discipline athletes being the tallest population (*p* < 0.0001), with the highest body weight (*p* < 0.0001) and the highest BSA (*p* < 0.0001) compared to other sports disciplines. Moreover, a higher DBP at rest was found in this group (*p* = 0.0001). Significant differences were also found in smoking habits (*p* < 0.0001), with no endurance athletes having this attitude whereas the highest prevalence was found in 15.1% of the athletes practicing skill disciplines and 13.4% of the mixed group. At CPET, significant differences among sport categories were found in multiple parameters (Table 2). A list of functional parameters (rest HR, maximal Watt, Watt/kg and O_2_ pulse and Watt and ${\dot{V}O}_{2}$ at first lactate threshold) showed an incremental significant increase (*p* < 0.0001) proceeding from sports with less aerobic components (skills) to those with maximal ones (endurance).

**Figure 1:**
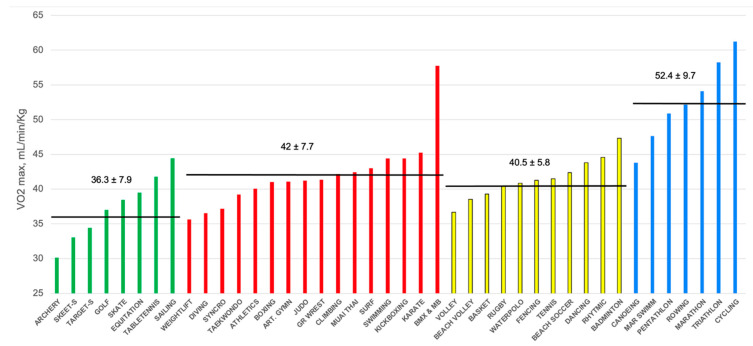
Values of ${\dot{V}O}_{2}$max in mL/min/Kg according to practiced sports discipline, divided into skill (green), power (red), mixed (yellow) and endurance (blue) categories.

For other functional parameters, a clear difference was noted for skill and endurance disciplines, but an overlap amount was noted between the mixed and power categories. In fact, athletes practicing mixed disciplines presented, for the following parameters, lower or similar data compared to power athletes. Specifically, METS peaks reached in exercise stress tests were higher in power (12 ± 2.3 vs. 11.5 ± 1.8 in mixed, *p* = 0.008) as well as ${\dot{V}O}_{2}$max (42 ± 7.7 mL/min/kg vs. 40.5 ± 5.8 mL/min/kg in mixed, *p* = 0.005). Moreover, no significant differences were found for VE max (100.9 ± 28 L/min, vs. 104.7 ± 27 L/min, *p* = 0.075), VT (2.58 ± 1.6 L in power vs. 2.68 ± 0.6 L in mixed, *p* = 0.307), Watts at second lactate threshold (205.4 ± 66.7 in power vs. 212.9 ± 76.7 in mixed, *p* = 0.191) and ${\dot{V}O}_{2}$ at second lactate threshold (2564.1 ± 752 mL/min in power vs. 2649.9 ± 905.6 mL/min in mixed, *p* = 0.199). Therefore, in order to evaluate the influence of a single sport discipline on these functional parameters, we have analyzed the ${\dot{V}O}_{2}$max values in all disciplines (Table 3). As also graphically shown in Figure 1, a certain quote of heterogeneity exists in all sport categories. Globally, similar values of ${\dot{V}O}_{2}$max were found in power and mixed disciplines, with BMX and mountain cyclists presenting values more suitable to the endurance category, as sailing (in skill) had values similar to mixed\power.

## 4. Discussion

The present study describes CPET values in a large cohort of 1033 elite athletes practicing different sports disciplines. Of interest, the main finding of the present study is that significant differences in CPET parameters exist among different sports disciplines, with endurance athletes showing the highest values of ${\dot{V}O}_{2}$/Kg, watt max and O_2_ pulse of the entire cohort, reflecting higher cardiovascular adaptation to physical exercise. To our knowledge, this is one of the largest cohorts in which comprehensive cardiopulmonary exercise testing has been systematically analyzed, providing robust normative data for this unique population. These differences likely reflect sport-specific cardiovascular and peripheral adaptations to chronic training stimuli, reinforcing the concept that athletic training induces highly divergent physiological remodeling depending on the nature and demands of the sport. At the same time, the novel information of this study is to provide, for the first time, values of CPET parameters derived from a large cohort of elite athletes according to the sports categories defined by the ESC sports classification [3]. We acknowledge that the analysis based on only four sports categories is flawed because differences even between sports of the same group may be profound. Nevertheless, in daily clinical practice, the ESC sport classification is largely implemented and is used even for the interpretation of cardiac sports adaptations. Our study adds a novel layer of functional data to this framework. The present investigation adds a new value to the ESC sport classification based mostly on cardiac dimensions originally derived from echocardiography [16], by supporting the morphologic differences observed among the four sport groups with functional data. However, the functional parameters derived from CPET in our study reflect the ESC classification only for skill and endurance sports. Indeed, a significant overlap was observed between mixed and power sports, suggesting that some disciplines currently classified as power sports should instead be considered mixed sports and vice versa. This observation supports the notion that the current classification, while practical, may oversimplify the heterogeneous demands of some disciplines. For example, BMX and mountain biking showed VO_2_/kg values that exceeded the mean plus two standard deviations of the power category, suggesting a markedly higher aerobic demand than expected for this group.

This finding aligns with prior research indicating that certain sports might require reclassification within the ESC categorization as well. Previous studies have actually demonstrated that some disciplines do not perfectly fit within their assigned category, highlighting the need for a more refined classification system [17,18,19,20,21]. Therefore, our investigation further supports the necessity of a revision of the current ESC classification that should also consider functional parameters and desirably reclassify some sports disciplines.

However, there is a paucity of data regarding CPET values among elite athletes. Most studies focus on sedentary individuals or healthy and physically active subjects but do not include elite or competitive athletes [22,23]. As such, Wagner et al. evaluated 502 healthy participants, analyzing the effects of physical activity. They directly measured ${\dot{V}O}_{2}$ peaks in healthy men and women over a wide age range (aged 20 to 90) and observed that the ${\dot{V}O}_{2}$ peak was higher when they performed moderate and vigorous-intensity physical activity [22].

Regarding athletes, most of the available studies on CPET are about children or adolescents [24,25]. Oleksak et al. [26] enrolled 136 healthy male Caucasian athletic children and adolescents (8–18 years old) from a Slovakian football school to provide reference values for CPET in this cohort. They noticed that resting and peak HR and VE/VCO_2_, as a marker of ventilatory effectivity, declined with age in highly active children. On the other hand, the resting O_2_ pulse and peak O_2_ pulse rose with age, as well as tidal volume, minute ventilation and ${\dot{V}O}_{2}$max, while resting breathing rate, ${\dot{V}O}_{2}$/WR, ${\dot{V}O}_{2}$ and % ${\dot{V}O}_{2}$ of ${\dot{V}O}_{2}$max in AT remained stable across childhood. This study presented only male athletes’ reference values and was set for a specific population, such as children/adolescents competing in a single sport.

A few studies were conducted on adult athletic populations, and none described the differences in CPET values based on sports categories and sex. Only Petek and colleagues focused on elite athletes [27]. They enrolled 272 athletes who underwent CPET, both on a treadmill and cycle ergometer, deriving and validating novel prediction equations for the ${\dot{V}O}_{2}$ peak to improve the diagnostic accuracy of CPET in the clinical care of athletes. They found slightly lower ${\dot{V}O}_{2}$ peak values among athletes tested on the cycle ergometer compared to our data. However, this study included a prevalence of endurance athletes, primarily cyclists, with a wide range of ages (18–74).

To sum up, reference ranges for CPET values in adult competitive or elite athletes do not currently exist in the literature. As an important consequence, the interpretation of CPET among athletes is based on general population references or limited data derived from studies including small sizes of athletes as subgroups of their populations, as well as some recent reviews on the highlighted topic [28,29,30].

Referring to general-population values when interpreting CPET results in elite athletes could generate a misinterpretation of parameters, especially among highly trained athletes, whose physiological adaptations to exercise may not correspond to normal values and result in unexpectedly impaired cardio-pulmonary fitness. At the same time, some parameters that would be considered abnormal for the general population would, instead, be just physiological among athletes [31,32,33].

Indeed, a comprehensive analysis of cardio-pulmonary fitness data could help to define better physiological cardiac adaptation to strenuous and continuous physical exercise and would then be of fundamental importance in the setting of early diagnosis of structural heart diseases and prevention of sudden cardiac death among young populations.

In summary, this study not only provides a comprehensive dataset of CPET parameters in elite athletes but also raises important considerations for both the scientific understanding of sport-specific physiological adaptations and the clinical interpretation of functional data in this population. The findings underscore the need to refine existing classification systems and to develop tailored reference standards that reflect the unique characteristics of elite athletes.

## 5. Limitations

Our study has some limitations. First, our population is predominantly Caucasian. Thus, CPET values derived from this cohort cannot be applied to other ethnic groups.

Next is the absence of a control group of the same age, gender and demographic characteristics to evaluate the variations in absolute values of CPET parameters and better assess the type and the magnitude of differences between athletes and the non-athletic population submitted to our screening protocol.

Furthermore, in our study, the classification of sports was based on both the ESC and COCIS guidelines, which may introduce variability in the categorization of certain disciplines.

In addition, in our study, CPET was performed exclusively on a cycle ergometer, which, although standardized, may not fully represent the peak functional capacity of athletes engaged in weight-bearing or non-cycling-based sports disciplines.

Moreover, this is a single-center study, and while it includes a large and diverse cohort of elite athletes, the findings should be interpreted within the context of this specific setting. Future multicenter studies could help validate and expand upon our findings.

Finally, although athletes were evaluated during a period of training, seasonal variability has not been specifically considered; therefore, it is reasonable that athletes adjust their training load throughout the season with possible fluctuations in CPET parameters.

## 6. Conclusions

This study provided mean values of CPET parameters in a large cohort of healthy elite athletes of both sexes and all sports categories. Significant differences in CPET parameters were observed among different sports disciplines, with endurance athletes showing the highest absolute and relative values in all parameters. An overlap amount was noted between the mixed and power categories.

Continuous research in this area will enhance our understanding of the physiological adaptations of elite athletes and contribute to the development of more effective diagnostic and preventive measures.

## Figures and Tables

**Table 1:** Demographic and clinical characteristics of the population according to sports discipline.

	Skill	Power	Mixed	Endurance	P Pooled	P Pairwise
N, (%)	146 (14.1)	343 (33.2)	344 (33.3)	200 (19.4)		
Age, years	26.4 ± 8.5	24.3 ± 4	26 ± 5	26.4 ± 3.9	<0.0001	**P vs. M, *p* < 0.0001; P vs. E, *p* < 0.0001; S vs. P, *p* = 0.0003**; S vs. M, *p* = 0.643; S vs. E, *p* = 0.973; M vs. E, *p* = 0.454.
Female, (%)	61 (41.8)	170 (49.6)	167 (48.5)	85 (42.5)	0.218	-
Afro-Caribbean, n (%)	0 (0)	27 (7.9)	12 (3.5)	8 (4)	0.0008	**S vs. P, *p* = 0.0005; S vs. M, *p* = 0.020; S vs. E, *p* = 0.014; P vs. M, *p* = 0.013;** P vs. E, *p* = 0.076; M vs. E, *p* = 0.760.
Height, cm	173.7 ± 8.7	173.6 ± 9.8	180.7 ± 11.9	177.7 ± 10	<0.0001	**S vs. M, *p* < 0.0001; S vs. E, *p* = 0.0001; P vs. M, *p* < 0.0001; P vs. E, *p* < 0.0001; M vs. E, *p* = 0.003;** S vs. P, *p* = 0.927.
Weight, kg	71.8 ± 14.2	70.1 ± 14.1	77.8 ± 14.2	71.6 ± 13.1	<0.0001	**S vs. M, *p* < 0.0001; P vs. M, *p* < 0.0001; M vs. E, *p* < 0.0001;** S vs. P, *p* = 0.236; S vs. E, *p* = 0.905; P vs. E, *p* = 0.228;
BMI, kg/m^2^	23.7 ± 3.8	23.1 ± 3.1	23.7 ± 2.4	22.5 ± 2.4	<0.0001	**S vs. E, *p* = 0.005; P vs. M, *p* = 0.006; P vs. E, *p* = 0.018; M vs. E, *p* < 0.0001;** S vs. P, *p* = 0.075; S vs. M, *p* = 0.968.
BSA	1.85 ± 0.21	1.82 ± 0.22	1.96 ± 0.24	1.87 ± 0.22	<0.0001	**S vs. M, *p* < 0.0001; P vs. M, *p* < 0.0001; P vs. E, *p* = 0.010;** **M vs. E, *p* < 0.0001;** S vs. E, *p* = 0.284; S vs. P, *p* = 0.241.
SBP, mmHg	114.5 ± 9.7	114.3 ± 10.7	115.8 ± 9.5	114.4 ± 10.1	0.180	-
DBP, mmHg	69 ± 7.3	69 ± 7.6	71 ± 7.2	69.2 ± 7	0.0001	**S vs. M, *p* = 0.013; P vs. M, *p* < 0.0001; M vs. E, *p* = 0.004;** S vs. P, *p* = 0.346; S vs. E, *p* = 0.984; P vs. E, *p* = 0.296.
Familiarity for CAD, n (%)	49 (33.6)	119 (34.7)	118 (34.3)	52 (26)	0.161	-
Smokers, n (%)	22 (15.1)	19 (5.5)	46 (13.4)	0 (0)	<0.0001	**S vs. P, *p* = 0.0005; S vs. E, *p* < 0.0001; P vs. M, *p* = 0.0004; P vs. E, *p* = 0.0007; M vs. E, *p* < 0.0001;** S vs. M, *p* = 0.620.

Abbreviations: BMI: body mass index; BSA: body surface area; CAD: coronary artery disease; DBP: diastolic blood pressure; SBP: systolic blood pressure.

**Table 2:** Comparison of CPET parameters according to sports discipline.

	Skill	Power	Mixed	Endurance	P Pooled	P Pairwise
N, (%)	146 (14.1)	343 (33.2)	344 (33.3)	200 (19.4)		
Rest HR, bpm	63.8 ± 11.1	58.3 ± 10.6	56.8 ± 9.2	52.1 ± 10	<0.0001	P vs. M, *p* = 0.048; remaining *p* < 0.0001
Peak HR, bpm	173.9 ± 12.5	169.4 ± 12.1	166 ± 12.1	166.1 ± 13.1	<0.0001	S vs. P, *p* = 0.0002; S vs. M, *p* < 0.0001; S vs. E, *p* < 0.0001; P vs. M, *p* = 0.0002; P vs. E, *p* = 0.002; M vs. E, *p* = 0.921.
MTHR, %	89.4 ± 4.9	85.9 ± 5.5	85.2 ± 5.3	85.1 ± 6.9	<0.0001	S vs. P, *p* < 0.0001; S vs. M, *p* < 0.0001; S vs. E, *p* < 0.0001; P vs. M, *p* = 0.078; P vs. E, *p* = 0.117; M vs. E, *p* = 0.817.
Watt max	197.9 ± 54.4	236.5 ± 62.3	252.1 ± 58.6	316.5 ± 94.3	<0.0001	All *p* < 0.0001.
Watt/Kg	2.79 ± 0.7	3.39 ± 0.8	3.23 ± 0.5	4.42 ± 1	<0.0001	All *p* < 0.0001.
METs Peak	10.3 ± 2.4	12 ± 2.3	11.5 ± 1.8	15.3 ± 6.3	<0.0001	P vs. M, *p* = 0.008; remaining *p* < 0.0001
Peak SBP, mmHg	172.1 ± 22	170.8 ± 17.6	176.5 ± 17.6	177.2 ± 20.3	<0.0001	S vs. M, *p* = 0.021; S vs. E, *p* = 0.028; P vs. M, *p* < 0.0001; P vs. E, *p* = 0.0002; M vs. E, *p* = 0.672; S vs. P, *p* = 0.500.
Peak DBP, mmHg	81.1 ± 7.7	79.6 ± 8.1	80.3 ± 7.5	79.4 ± 6.8	0.115	-
${\dot{V}O}_{2}$max, mL/min/kg	36.3 ± 7.9	42 ± 7.7	40.5 ± 5.8	52.4 ± 9.7	<0.0001	P vs. M, *p* = 0.005; remaining *p* < 0.0001
${\dot{V}O}_{2}$/Watt	13.1 ± 1.9	12.5 ± 1.5	12.6 ± 1.6	12.6 ± 7	0.427	-
VCO_2_, mL/min	2907.1 ± 709.5	3219.3 ± 839.5	3439 ± 763.9	3980 ± 989.3	<0.0001	P vs. M, *p* = 0.0004; remaining *p* < 0.0001.
RQ	1.15 ± 0.08	1.12 ± 0.08	1.11 ± 0.07	1.08 ± 0.08	<0.0001	P vs. M, *p* = 0.231; remaining *p* < 0.0001.
O_2_ pulse, mL/beat	14.9 ± 3.8	17.5 ± 4.6	19 ± 4.3	22.7 ± 5.8	<0.0001	All *p* < 0.0001
VE max, L/min	90.7 ± 20.7	100.9 ± 28	104.7 ± 27	121.3 ± 33.7	<0.0001	P vs. M, *p* = 0.075; remaining *p* < 0.0001.
VT, L	2.41 ± 0.6	2.58 ± 1.6	2.68 ± 0.6	2.82 ± 0.7	<0.0001	S vs. M, *p* < 0.0001; S vs. E, *p* < 0.0001; M vs. E, *p* < 0.0001; P vs. M, *p* = 0.307; S vs. P, *p* = 0.225; P vs. E, *p* = 0.053.
Watt @ LT1	104.1 ± 41	125.2 ± 47	134.2 ± 43.2	203.2 ± 93.6	<0.0001	All *p* < 0.0001
${\dot{V}O}_{2}$@ LT1	1543.1 ± 484.8	1756 ± 533.1	1869.5 ± 505.4	2563.3 ± 978.6	<0.0001	P vs. M, *p* = 0.004; remaining *p* < 0.0001.
VE/VCO_2_ @ LT1	26.5 ± 2.8	26.4 ± 2.8	26.1 ± 3.1	27 ± 2.9	0.031	P vs. E, *p* = 0.018; M vs. E, *p* = 0.005; S vs. P, *p* = 0.664; S vs. M, *p* = 0.334; S vs. E, *p* = 0.130; P vs. M, *p* = 0.463.
Watt @ LT2	168.9 ± 56.2	205.4 ± 66.7	212.9 ± 76.7	275.4 ± 103.7	<0.0001	P vs. M, *p* = 0.191; remaining *p* < 0.0001.
${\dot{V}O}_{2}$@ LT2	2279 ± 1066.8	2564.1 ± 752	2649.9 ± 905.6	3248.5 ± 1070.2	<0.0001	S vs. P, *p* = 0.001; S vs. M, *p* = 0.0002; S vs. E, *p* < 0.0001; P vs. M, *p* = 0.199; P vs. E, *p* < 0.0001; M vs. E, *p* < 0.0001.
VE/VCO_2_ @ LT2	27.6 ± 3.3	27.7 ± 3.2	27 ± 3.2	27.9 ± 3.4	0.014	P vs. M, *p* = 0.007; M vs. E, *p* = 0.006; S vs. P, *p* = 0.637; S vs. M, *p* = 0.103; S vs. E, *p* = 0.366; P vs. E, *p* = 0.534.

Abbreviations: DBP: diastolic blood pressure; HR: heart rate; LT1: first lactate threshold; LT2: second lactate threshold; MET: metabolic equivalent of task; MTHR: maximal theoretical heart rate; RQ: respiratory quotient; SBP: systolic blood pressure; VE: ventilation; VE/VCO_2_: ventilatory efficiency; VT: tidal volume.

**Table 3:** ${\dot{V}O}_{2}$max values in specific sports disciplines divided according to 2020 ESC/COCIS classification.

Sport Category	Sport Discipline	N, (%)	${\dot{V}O}_{2}$max, mL/min/Kg
Skill 146 (14.1%)	Archery	15 (10.3)	30.1 ± 4.9
	Skeet shooting	29 (19.9)	33 ± 11
	Target shooting	19 (13)	34.4 ± 5.9
	Golf	43 (29.4)	37 ± 5.9
	Park and street skateboarding	5 (3.4)	38.4 ± 9
	Equitation	18 (12.3)	39.5 ± 3.9
	Table tennis	5 (3.4)	41.8 ± 5.2
	Sailing	12 (8.2)	44.5 ± 5.6
Power 343 (33.2%)	Weightlifting	7 (2)	35.6 ± 5.8
	Diving	16 (4.7)	36.6 ± 4.8
	Synchronized swimming	19 (5.5)	37.2 ± 3.1
	Taekwondo	13 (3.8)	39.2 ± 6.8
	Athletics (<800 mt)	67 (19.5)	40 ± 6.5
	Boxing	20 (5.8)	41 ± 6.5
	Artistic gymnastics	24 (7)	41.1 ± 4.3
	Judo	32 (9.3)	41.3 ± 6.4
	Greek–Roman wrestling	21 (6.1)	41.4 ± 6.0
	Climbing	20 (5.8)	42.2 ± 6.6
	Muai Thai	4 (1.1)	42.4 ± 4
	Surfing	4 (1.1)	43 ± 8.4
	Swimming	54 (15.7)	44.4 ± 7.5
	Kickboxing	14 (4.1)	44.4 ± 5
	Karate	11 (3.2)	45.2 ± 5.3
	BMX and mountain biking	17 (5)	57.7 ± 11.7
Mixed 344 (33.3%)	Volleyball	66 (19.2)	36.7 ± 5.2
	Beach volleyball	6 (1.7)	38.5 ± 5.1
	Basketball	31 (9)	39.3 ± 5.5
	Rugby	36 (10.5)	40.4 ± 3.9
	Water polo	61 (17.7)	40.9 ± 3.9
	Fencing	40 (11.6)	41.3 ± 5.1
	Tennis and paddle	22 (6.4)	41.5 ± 5.5
	Beach soccer	46 (13.4)	42.4 ± 5.8
	Sporting dancing	13 (3.8)	43.8 ± 5.4
	Rhythmic gymnastics	15 (4.4)	44.6 ± 4.7
	Badminton	8 (2.3)	47.3 ± 7.2
Endurance 200 (19.4%)	Canoeing	46 (23)	43.8 ± 5.3
	Marathon swimming	8 (4)	47.7 ± 9
	Pentathlon	17 (8.5)	50.9 ± 3.9
	Rowing	47 (23.5)	52.1 ± 6.9
	Marathon running	32 (16)	54.1 ± 10.6
	Triathlon	13 (6.5)	58.2 ± 10.8
	Cycling	37 (18.5)	61.2 ± 8.7

## Data Availability

Underlying data not included in this article are available from the corresponding author upon reasonable request.
